# MicroRNA-155 contributes to shear-resistant leukocyte adhesion to human brain endothelium in vitro

**DOI:** 10.1186/s12987-016-0032-3

**Published:** 2016-05-31

**Authors:** Camilla Cerutti, Patricia Soblechero-Martin, Dongsheng Wu, Miguel Alejandro Lopez-Ramirez, Helga de Vries, Basil Sharrack, David Kingsley Male, Ignacio Andres Romero

**Affiliations:** Department of Life, Health and Chemical Sciences, Biomedical Research Network, Open University, Walton Hall, Milton Keynes, MK7 6AA UK; Department of Neuroscience, Sheffield University, 385a Glossop Road, Sheffield, S10 2HQ UK; Randall Division of Cell and Molecular Biophysics, King’s College London, New Hunt’s House, Guy’s Campus, London, SE1 1UL UK; School of Engineering and Materials Science, Queen Mary University of London, Mile End Road, London, E1 4NS UK; Department of Medicine, University of California, San Diego, La Jolla, CA 92093 USA; Department of Molecular Cell Biology and Immunology, MS Centre Amsterdam, VU University Medical Centre, Amsterdam, The Netherlands

**Keywords:** Blood–brain barrier, Cell adhesion molecules, Flow shear stress, Leukocyte adhesion, microRNA-155, Neuroinflammation

## Abstract

**Background:**

Increased leukocyte adhesion to brain endothelial cells forming the blood–brain barrier (BBB) precedes extravasation into the central nervous system (CNS) in neuroinflammatory diseases such as multiple sclerosis (MS). Previously, we reported that microRNA-155 (miR-155) is up-regulated in MS and by inflammatory cytokines in human brain endothelium, with consequent modulation of endothelial paracellular permeability. Here, we investigated the role of endothelial miR-155 in leukocyte adhesion to the human cerebral microvascular endothelial cell line, hCMEC/D3, under shear forces mimicking blood flow in vivo.

**Results:**

Using a gain- and loss-of-function approach, we show that miR-155 up-regulation increases leukocyte firm adhesion of both monocyte and T cells to hCMEC/D3 cells. Inhibition of endogenous endothelial miR-155 reduced monocytic and T cell firm adhesion to naïve and cytokines-induced human brain endothelium. Furthermore, this effect is partially associated with modulation of the endothelial cell adhesion molecules VCAM1 and ICAM1 by miR-155.

**Conclusions:**

Our results suggest that endothelial miR-155 contribute to the regulation of leukocyte adhesion at the inflamed BBB. Taken together with previous observations, brain endothelial miR-155 may constitute a potential molecular target for treatment of neuroinflammation diseases.

**Electronic supplementary material:**

The online version of this article (doi:10.1186/s12987-016-0032-3) contains supplementary material, which is available to authorized users.

## Background

Leukocyte recruitment from blood into tissues is a crucial event in both physiological and pathological conditions and is described as a multistep process involving leukocyte rolling, adhesion, crawling and diapedesis [1] under hemodynamic shear stress. In the central nervous system (CNS), firm leukocyte adhesion to the highly specialized brain endothelial cells forming the blood–brain barrier (BBB) is important in immunosurveillance and plays a critical role in the pathogenesis of neuroinflammatory diseases such as multiple sclerosis (MS) [2]. Leukocyte adhesion occurs in postcapillary venules [3] as a result of specific interactions between leukocyte integrins, α4β1 (VLA-4) and αLβ2-integrin (LFA-1), and endothelial adhesion molecules, VCAM1 and ICAM1, respectively [4]. In MS, chemokines and proinflammatory cytokines such as TNFα and IFNγ are secreted in the inflammatory loci thereby leading to VCAM1 and ICAM1 overexpression on activated brain endothelial cells [2]. Furthermore, it has been observed that both monocytes and T cells are present in the perivascular inflammatory infiltrates [5]. Despite numerous studies, the endothelial molecular controls on leukocyte firm adhesion to brain endothelium have not been fully elucidated.

MicroRNAs (miRs) are a class of highly conserved, single-stranded, non-coding RNA molecules (20–25 nucleotides), that modulate gene expression by repression of their target genes at the post-transcriptional level [6]. Recent studies have identified miRs as key regulators of a vast number of biological processes and disorders, including MS [7] and those regulating neurovascular function in inflammation [8], such as regulation of cell adhesion molecules and leukocyte trafficking across brain endothelium [9, 10].

MiR-155 is a multifunctional miR which plays a crucial role in physiological and pathological processes including inflammation [11, 12]. MiR-155 expression is increased in brain endothelium in MS active lesions and proinflammatory cytokines, TNFα and IFNγ, up-regulate miR-155 expression in the human cerebral microvascular endothelial cell line, hCMEC/D3 [13]. Furthermore, miR-155 overexpression in hCMEC/D3 cells increases endothelial permeability and negatively affects expression of tight junctional molecules, whereas miR-155 inhibition is associated with decreased microvascular permeability [13]. In this study, we determined the role of human brain endothelial miR-155 in controlling T cell and monocyte firm adhesion to hCMEC/D3 cells, an in vitro model of human brain endothelium [14], when subjected to shear forces mimicking blood flow at the venular vessel level in vivo. In addition, the effect of miR-155 on the expression of the cell adhesion molecules VCAM1 and ICAM1 in hCMEC/D3 cells was also investigated.

## Methods

### Cell culture

The hCMEC/D3 cell line [14] was used at passages 26–34 and cultured in endothelial cell basal medium-2 (EGM-2) medium (Lonza, Walkersville, USA) and supplemented with the following components obtained from the manufacturer: 0.025 % (v/v) rhEGF, 0.025 % (v/v) VEGF, 0.025 % (v/v) IGF, 0.1 % (v/v) rhFGF, 0.1 % (v/v) gentamycin, 0.1 % (v/v) ascorbic acid, 0.04 % (v/v) hydrocortisone and 2.5 % (v/v) foetal bovine serum (FBS), hereafter referred to as endothelial complete medium. hCMEC/D3 cells were grown to confluence (~1 × 10^5^ cells/cm^2^) on tissue culture flasks coated with collagen from calf skin (Sigma, St. Louis, USA). The T cell line Jurkat from acute T cell leukaemia and the monocytic line THP1 from acute monocytic leukaemia were a kind gift from Dr. V Male (Cambridge University). Jurkat and THP1 cells were grown in suspension in RPMI 1640 W/GLUTAMAX I (Gibco^®^Invitrogen, Paisley,UK) culture medium (containing 10 % FBS and 100 μg/ml streptomycin + 100 units/ml penicillin). All cell lines were maintained in a 95 % humidified air and 5 % CO_2_ incubator at 37 °C.

### MicroRNA transfection

hCMEC/D3 cells were grown to ~70 % confluence and transfected in antibiotic-free endothelial media. To introduce miR-155 precursor, hCMEC/D3 cells were transfected with 30 nM of pre-miR-155 or its control, scrambled-pre-miR (Ambion, Fischer Scientific UK), using Siport™ Polyamine Transfection Agent (Ambion) in Opti-mem^®^I (Gibco^**®**^) media for 24 h. For inhibition studies, 60 nM of anti-miR-155 or its control, scrambled-anti-miR (Dharmacon, Waltham, USA) was transfected using Lipofectamine^®^ 2000 (Thermo Fisher Scientific, Carlsbad, USA) for 6 h, media was then changed with endothelial complete medium for 18 h. siGENOME SMARTpool siRNAs for human VCAM1 or siRNA control pool (ThermoFisher Scientific) were transfected into hCMEC/D3 cells using Lipofectamine 2000^®^ (Thermo Fisher Scientific).

### Flow-based leukocyte adhesion assay: live cell adhesion imaging under flow conditions

A flow-based adhesion assay previously described in Wu et al. was used [10]. hCMEC/D3 cells were grown in Ibidi^®^ μ-Slide VI^0.4^ (Ibidi^®^ GmbH, Martinstreid, Germany), transfected, treated or not with 1 ng/ml TNFα and IFNα for 24 h in static conditions and washed before flow adhesion assay. THP-1 and Jurkat cells (2 × 10^6^ cells/ml) were labelled with CMFDA (5–chloromethylfluoresceindiacetate, Life Technologies, Eugene, USA) and were allowed to flow through the channel with endothelial monolayers and accumulate at 0.5 dyn/cm^2^ for 5 min. Then, the flow was increased to 1.5 dyn/cm^2^ (venular vessel wall shear stress) for 30 s to remove non-adhered leukocytes with endothelial complete media. Leukocyte-endothelial interactions were recorded (Additional file 1: Video S1) for 5.5 min and firm leukocyte adhesion was quantified. Firm adhesion was defined by leukocytes that remained adhered on human brain endothelium in the field of view (FOV 640 × 480 μm) throughout the accumulation time and after increasing the flow to 1.5 dyn/cm^2^ and manually counted using Image J software in five different FOVs. Image acquisition was performed using a X10 objective of an inverted fluorescence microscope (Olympus IX70, Tokyo, Japan) controlled by the Image Pro Plus software (Media Cybernetics Inc. Bethesda, USA) using a Q-IMAGING *QICAM FAST* 1394 on a 12-bit camera (40 images/min). For more details refer to Additional file 2: Fig. S1, Table S1 and Table S2.

### ELISA for adhesion molecules

Brain endothelial expression of VCAM1 and ICAM1 was measured by cell-surface ELISA performed as previously described [15] using 2 μg/ml mouse primary antibody against VCAM1 or ICAM1 (R&D SYSTEMS, Abingdon, UK) and the corresponding secondary antibodies conjugated to horseradish peroxidase. The optical density (OD) was then measured using a FLUOstar Optima spectrometer (BMG LABTECH, Aylesbury, UK) at a wavelength of 450 nm.

### Statistics

All data are presented as mean ± SEM from a number of independent experiments (n) with replicates specified in each legend. *P* values were calculated using paired Student’s *t* tests. Statistically significant differences are presented as probability levels of *P* < 0.05 (*), *P* < 0.01 (**). Calculations and figures were performed using the statistical software GraphPad Prism 5 (GraphPad Software).

## Results

### MiR-155 modulates Jurkat and THP-1 firm adhesion to hCMEC/D3 cells

**Fig. 1:**
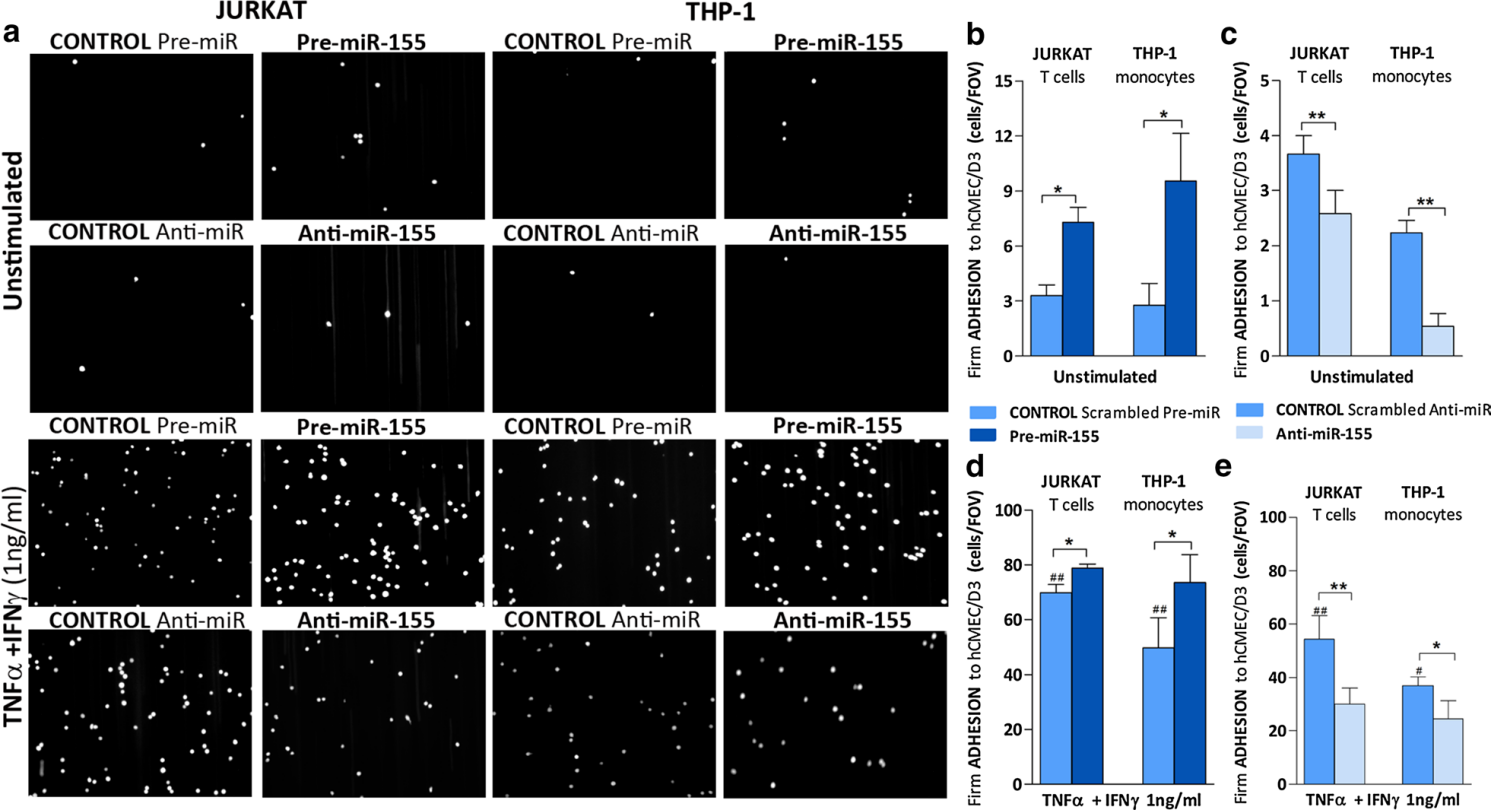
miR-155 modulates Jurkat and THP-1 firm adhesion to brain endothelial hCMEC/D3 cells. hCMEC/D3 cell monolayers were transfected with control scrambled Pre-miR and Pre-miR-155 (**a**, **c**, **d**) or control scrambled Anti-miR and Anti-miR-155 (**a**, **c** , **e**) followed by treatment with a combination of cytokines (TNFα + IFNγ) at 1 ng/ml for 24 h (**a**, **d** , **e**) or left unstimulated (**a**, **b** , **c**). **a** Representative images of shear-resistant firmly adhered Jurkat and THP-1 cells to hCMEC/D3 monolayer (field of view (FOV): 640 × 480 μm) used for quantification and **b**-**e** analysis of shear-resistant firmly adhered Jurkat and THP-1 cells to hCMEC/D3 expressed in number of cells/FOV. Experiments were carried out three to six times with five FOVs each. Data are mean ± SEM. Statistical analysis was performed using paired Student’s *t test* (^*,#^ *P* < 0.05, ^**,##^ *P* < 0.01, ^#^compared to unstimulated)

We first investigated whether increased levels of miR-155 in unstimulated brain endothelial cells affected firm leukocyte adhesion under shear stress. In human brain endothelium, miR-155 overexpression simulates, to a certain extent, the effect of proinflammatory cytokines [13], which are known to increase T cell firm adhesion [10]. We observed strong increase in adhesion of both T cell (Jurkat ~twofold increase) and monocyte (THP-1 ~threefold increase) to unstimulated hCMEC/D3 cells transfected with miR-155 precursor (pre-miR-155) compared with control (scrambled pre-miR) (Fig. 1a, b; Additional file 3: Video S2, Additional file 4: Video S3, Additional file 5: Video S4, Additional file 6: Video S5). Inhibition of endogenous miR-155 in hCMEC/D3 cells by transfection with anti-miR-155 reduced Jurkat and THP-1 firm adhesion to unstimulated brain endothelium compared to its control (scrambled anti-miR) (Fig. 1a and c; Additional file 7: Video S6, Additional file 8: Video S7, Additional file 9: Video S8, Additional file 10: Video S9). To better understand the contribution of endothelial miR-155 in leukocyte adhesion, in the context of inflammation, we then explored the effect of miR-155 modulation on monocytic and T cell adhesion on brain endothelial cells stimulated with pro-inflammatory cytokines (TNFα and IFNγ at 1 ng/ml for 24 h), a treatment that increases brain endothelial miR-155 expression, hence monocytic and T cell adhesion (Fig. 1d, e; controls). Over-expression of miR-155 slightly increased shear resistant leukocyte adhesion to cytokine-treated brain endothelium compared to control (cytokine-treated scrambled pre-miR) (Fig. 1 a, d; Additional file 11: Video S10, Additional file 12: Video S11, Additional file 13: Video S12, Additional file 14: Video S13). Reduction of endogenous miR-155 reduced monocytic and T cell adhesion by ~50 and ~35 %, respectively, to cytokine-stimulated endothelial cells when compared to control (cytokine-treated scrambled anti-miR) (Fig. 1a and e; Additional file 15: Video S14, Additional file 16: Video S15, Additional file 17: Video S16, Additional file 18: Video S17).

### MiR-155 modulates expression of cell adhesion molecules in hCMEC/D3 cells

**Fig. 2:**
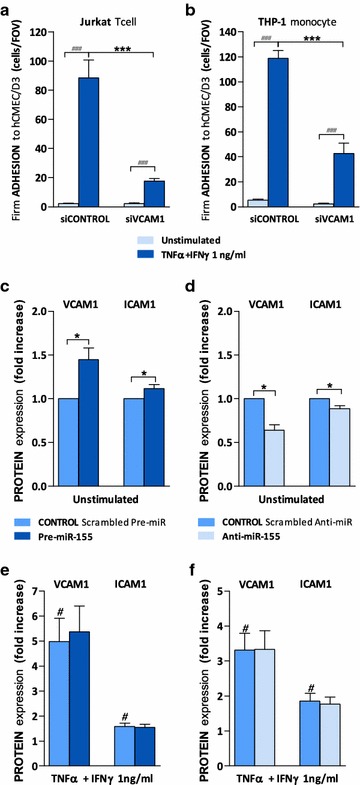
miR-155 modulates VCAM1 and ICAM1 expression on brain endothelial hCMEC/D3 cells. hCMEC/D3 cell monolayers were transfected with control siRNA (**a**, **b**) or scrambled Pre-miR and Pre-miR-155 (**c**, **e**) or Anti-miR and Anti-miR-155 (**d**, **f**) followed by treatment with a combination of cytokines (TNFα + IFNγ) at 1 ng/ml for 24 h or left unstimulated. **a**, **b** Number of shear-resistant firmly adhered Jurkat and THP-1 cells to siVCAM1-hCMEC/D3 monolayer per FOV (640 × 480 μm). **c**–**f** VCAM1 and ICAM1 expression levels were quantified by ELISA. Experiments were carried out three and four times with three replicates each. Data are mean ± SEM. Statistical analysis was performed using paired Student’s *t test* (^*,#^ *P* < 0.05, ^***,###^ *P* < 0.001, ^#^compared to unstimulated)

To further elucidate the role of miR-155 in leukocyte adhesion, we explored whether miR-155-induced changes in monocyte and T cell adhesion to endothelium were associated with modulation of cell adhesion molecules VCAM1 and ICAM1 on the endothelial surface, master mediators of leukocyte trafficking to the BBB [16]. ICAM1 plays a critical role in T cell adhesion as previously demonstrated [17], but VCAM1 is a main player in leukocyte adhesion and mediated both monocyte and T cells adhesion to brain endothelium (Fig. 2a, b). Overexpression of miR-155 enhanced VCAM1 and ICAM1 levels on unstimulated hCMEC/D3 cells (Fig. 2c) whereas decreasing the levels of miR-155 caused a small reduction in VCAM1 and ICAM1 expression (Fig. 2d). No changes in VCAM1 or ICAM1 expression by miR-155 were observed in cytokine-stimulated endothelium (Fig. 2e, f).

## Discussion

MiR-155 is strongly upregulated in cytokine-stimulated hCMEC/D3 cells and in EAE spinal cord vessels at acute stages of the disease, when the BBB is compromised [13]. The same study found that miR-155 acts as a novel regulator of barrier permeability by affecting expression of genes involved in modulation of tight junctions and cell to matrix interactions in human brain endothelium. In this study, we show that modulation of brain endothelial miR-155 levels led to significant changes on firm T cell and monocytic cell line adhesion to hCMEC/D3 cells. However, miR-155 induction of ICAM1 and VCAM1 endothelial expression, while significant, was relatively small in unstimulated conditions, and, no changes in CAM expression by miR-155 were observed in cytokine-treated cells. Therefore we consider that modulation of leukocyte adhesion to brain endothelium by endothelial miR-155 can only be partly accounted for by its effects in the expression of these adhesion molecules, in particular in the early stages of inflammation as miR-155 is one of the earliest microRNAs to be rapidly induced following inflammatory stimuli [13]. Indeed, increased levels of miR-155 enhanced by two fold the expression of two other adhesion-related genes, CCL5 and TNFSF10 in hCMEC/D3 cells (Geo accession GSE44694, platform GPL6883).

Indirect mechanisms other than directly regulating expression of cell adhesion molecules could account for the effect of endothelial miR-155 on leukocyte firm-adhesion. MiRs act by suppressing the expression of genes that contain the miR-target sequence in their mRNA and hence they directly reduce protein expression. Therefore, in order to modulate leukocyte adhesion, miR-155 may regulate the expression of genes which control adhesion indirectly. In this context, it is possible that miR-155 could target NFκB pathway in brain endothelium as it does in HUVEC [18]. This pathway is activated by TNFα leading to the phosphorylation and breakdown of IκB which releases NFκB, allowing it to enter the nucleus and activate several genes involved in neuroinflammation, including VCAM1 and ICAM1. IκB, the inhibitor of NFκB does not contain target sites for miR-155, but ‘Inhibitor of nuclear factor kappa-B kinase-interacting protein’ (IKBIP) is a potential target for miR-155 (Diana Tools, miRTarbase), previously validated by proteomics [19]. It is therefore conceivable that a reduction in IKBIP expression due to cytokine-induced miR-155 would promote IκB kinase (IKK) to mediate phosphorylation and degradation of IκB, thereby leading to increased nuclear translocation of NFκB, with wide-ranging down-stream effects including the one resulting in increased leukocyte adhesion. This goes hand in hand with our previous observation where inhibition of RelA, NFκB associated protein crucial for NFκB nuclear translocation and activation, decreased T cell adhesion by 60 % to hCMEC/D3 cells [10].

Another possible mechanism by which endothelial miR-155 may modulate leukocyte adhesion involves the small GTPase RhoA, a validated target of miR-155 [20]. Indeed, RhoA controls Rho-associated kinase (ROCK) which in turn modulates ICAM1 expression, cell adhesion, the NFκB pathway [21]. In addition, RhoA is thought to affect leukocyte adhesion and migration by its actions in controlling the organisation of the brain endothelial cytoskeleton [22]. In hCMEC/D3, reduced levels of RhoA induced decreased VCAM1 expression and T cell adhesion [10]. It is certainly possible that miR-155 targets more than one gene controlling either leukocyte adhesion or endothelial activation, and the two genes discussed here both have several important down-stream effects in controlling molecules involved in neuroinflammation and leukocyte adhesion.

## Conclusions

Taken together, our findings support the notion that in neuroinflammatory conditions, miR-155 is itself up-regulated and can promote many pro-inflammatory processes including leukocyte adhesion to brain endothelium. Because of their multiple effects on cellular processes, targeting an individual miR such as miR-155 for therapeutic purposes may lead to modulation of different activation pathways that promote inflammation. Together, these results reinforce the role of endothelial miR-155 in the pathophysiology of the BBB, with a wide range of pro-inflammatory effects.

## Additional files

10.1186/s13068-016-0531-0 Representative video of flow-based leukocyte adhesion assay using live cell imaging. Brightfield (Left Panel) and FITC (Right Panel) are displayed in parallel to show that the flow-based leukocyte adhesion assay used allows to perform live cell imaging recording both the endothelial monolayer by light microscopy and the fluorescent labelled leukocytes by fluorescent microscopy. The number of arrested JURKAT cells constantly increased along the channel of Ibidi^®^ Slide VI, the yellow arrows (Right Panel) indicate arrested Jurkat cells. Objective 10x, phase-contrast illumination and FITC channel (1 phase-contrast and 1 FITC), field of view 640 μm × 480 μm.
10.1186/s13068-016-0531-0 Fig. S1, Table S1, Table S2.
10.1186/s13068-016-0531-0 Shear-resistant firm adhesion of JURKAT cells to CONTROL Scrambled-Pre-miR transfected and unstimulated hCMECD3 cells. JURKAT cells were pulled through the channel over hCMEC/D3 monolayers under low shear (0.5 dyn/cm^2^). After 5 min, flow shear stress was increased (1.5 dyn/cm^2^) to challenge non-firmly adherent JURKAT. The number of arrested JURKAT constantly increased during the accumulation phase and only the one that remained stationary on the endothelial monolayer were manually counted in 5 different FOV along the channel of Ibidi m-Slide VI. FITC channel was used to count fluorescently firmly adhered Jurkat cells. Objective 10x, at 40 images per min (1 phase-contrast and 1 FITC), field of view 640 μm × 480 μm, recording time 5.5 min.
10.1186/s13068-016-0531-0 Shear-resistant firm adhesion of JURKAT cells to Pre-miR-155 transfected and unstimulated hCMECD3 cells. JURKAT cells were pulled through the channel over hCMEC/D3 monolayers under low shear (0.5 dyn/cm^2^). After 5 min, flow shear stress was increased (1.5 dyn/cm^2^) to challenge non-firmly adherent JURKAT. The number of arrested JURKAT constantly increased during the accumulation phase and only the one that remained stationary on the endothelial monolayer were manually counted in 5 different FOV along the channel of Ibidi m-Slide VI. FITC channel was used to count fluorescently firmly adhered Jurkat cells. Objective 10x, at 40 images per min (1 phase-contrast and 1 FITC), field of view 640 μm × 480 μm, recording time 5.5 min.
10.1186/s13068-016-0531-0 Shear-resistant firm adhesion of THP1 cells to CONTROL Scrambled-Pre-miR transfected and unstimulated hCMECD3 cells. THP1 cells were pulled through the channel over hCMEC/D3 monolayers under low shear (0.5 dyn/cm^2^). After 5 min, flow shear stress was increased (1.5 dyn/cm^2^) to challenge non-firmly adherent THP1. The number of arrested THP1 constantly increased during the accumulation phase and only the one that remained stationary on the endothelial monolayer were manually counted in 5 different FOV along the channel of Ibidi m-Slide VI. FITC channel was used to count fluorescently firmly adhered THP1 cells. Objective 10x, at 40 images per min (1 phase-contrast and 1 FITC), field of view 640 μm × 480 μm, recording time 5.5 min.
10.1186/s13068-016-0531-0 Shear-resistant firm adhesion of THP1 cells to Pre-miR-155 transfected and unstimulated hCMECD3 cells. THP1 cells were pulled through the channel over hCMEC/D3 monolayers under low shear (0.5 dyn/cm^2^). After 5 min, flow shear stress was increased (1.5 dyn/cm^2^) to challenge non-firmly adherent THP1. The number of arrested THP1 constantly increased during the accumulation phase and only the one that remained stationary on the endothelial monolayer were manually counted in 5 different FOV along the channel of Ibidi m-Slide VI. FITC channel was used to count fluorescently firmly adhered THP1 cells. Objective 10x, at 40 images per min (1 phase-contrast and 1 FITC), field of view 640 μm × 480 μm, recording time 5.5 min.
10.1186/s13068-016-0531-0 Shear-resistant firm adhesion of JURKAT cells to CONTROL Scrambled-Anti-miR transfected and unstimulated hCMECD3 cells. JURKAT cells were pulled through the channel over hCMEC/D3 monolayers under low shear (0.5 dyn/cm^2^). After 5 min, flow shear stress was increased (1.5 dyn/cm^2^) to challenge non-firmly adherent JURKAT. The number of arrested JURKAT constantly increased during the accumulation phase and only the one that remained stationary on the endothelial monolayer were manually counted in 5 different FOV along the channel of Ibidi m-Slide VI. FITC channel was used to count fluorescently firmly adhered Jurkat cells. Objective 10x, at 40 images per min (1 phase-contrast and 1 FITC), field of view 640 μm × 480 μm, recording time 5.5 min.
10.1186/s13068-016-0531-0 Shear-resistant firm adhesion of JURKAT cells to Anti-miR-155 transfected and unstimulated hCMECD3 cells. JURKAT cells were pulled through the channel over hCMEC/D3 monolayers under low shear (0.5 dyn/cm^2^). After 5 min, flow shear stress was increased (1.5 dyn/cm^2^) to challenge non-firmly adherent JURKAT. The number of arrested JURKAT constantly increased during the accumulation phase and only the one that remained stationary on the endothelial monolayer were manually counted in 5 different FOV along the channel of Ibidi m-Slide VI. FITC channel was used to count fluorescently firmly adhered Jurkat cells. Objective 10x, at 40 images per min (1 phase-contrast and 1 FITC), field of view 640 μm × 480 μm, recording time 5.5 min.
10.1186/s13068-016-0531-0 Shear-resistant firm adhesion of THP1 cells to CONTROL Scrambled-Anti-miR transfected and unstimulated hCMECD3 cells. THP1 cells were pulled through the channel over hCMEC/D3 monolayers under low shear (0.5 dyn/cm^2^). After 5 min, flow shear stress was increased (1.5 dyn/cm^2^) to challenge non-firmly adherent THP1. The number of arrested THP1 constantly increased during the accumulation phase and only the one that remained stationary on the endothelial monolayer were manually counted in 5 different FOV along the channel of Ibidi m-Slide VI. FITC channel was used to count fluorescently firmly adhered THP1 cells. Objective 10x, at 40 images per min (1 phase-contrast and 1 FITC), field of view 640 μm × 480 μm, recording time 5.5 min.
10.1186/s13068-016-0531-0 Shear-resistant firm adhesion of THP1 cells to Anti-miR-155 unstimulated transfected and unstimulated hCMECD3 cells. THP1 cells were pulled through the channel over hCMEC/D3 monolayers under low shear (0.5 dyn/cm^2^). After 5 min, flow shear stress was increased (1.5 dyn/cm^2^) to challenge non-firmly adherent THP1. The number of arrested THP1 constantly increased during the accumulation phase and only the one that remained stationary on the endothelial monolayer were manually counted in 5 different FOV along the channel of Ibidi m-Slide VI. FITC channel was used to count fluorescently firmly adhered THP1cells. Objective 10x, at 40 images per min (1 phase-contrast and 1 FITC), field of view 640 μm × 480 μm, recording time 5.5 min.
10.1186/s13068-016-0531-0 Shear-resistant firm adhesion of JURKAT cells to CONTROL Scrambled-Pre-miR transfected and stimulated with TNFα and IFNγ hCMECD3 cells. JURKAT cells were pulled through the channel over hCMEC/D3 monolayers under low shear (0.5 dyn/cm^2^). After 5 min, flow shear stress was increased (1.5 dyn/cm^2^) to challenge non-firmly adherent JURKAT. The number of arrested JURKAT constantly increased during the accumulation phase and only the one thatremained stationary on the endothelial monolayer were manually counted in 5 different FOV along the channel of Ibidi m-Slide VI. FITC channel was used to count fluorescently firmly adhered Jurkat cells. Objective 10x, at 40 images per min (1 phase-contrast and 1 FITC), field of view 640 μm × 480 μm, recording time 5.5 min.
10.1186/s13068-016-0531-0 Shear-resistant firm adhesion of THP1 cells to CONTROL Scrambled-Pre-miR transfected and stimulated with TNFα and IFNγ hCMECD3 cells. THP1 cells were pulled through the channel over hCMEC/D3 monolayers under low shear (0.5 dyn/cm^2^). After 5 min, flow shear stress was increased (1.5 dyn/cm^2^) to challenge non-firmly adherent THP1. The number of arrested THP1 constantly increased during the accumulation phase and only the one that remained stationary on the endothelial monolayer were manually counted in 5 different FOV along the channel of Ibidi m-Slide VI. FITC channel was used to count fluorescently firmly adhered THP1 cells. Objective 10x, at 40 images per min (1 phase-contrast and 1 FITC), field of view 640 μm × 480 μm, recording time 5.5 min.
10.1186/s13068-016-0531-0 Shear-resistant firm adhesion of THP1 cells to Pre-miR-155 transfected and stimulated with TNFα and IFNγ hCMECD3 cells. THP1 cells were pulled through the channel over hCMEC/D3 monolayers under low shear (0.5 dyn/cm^2^). After 5 min, flow shear stress was increased (1.5 dyn/cm^2^) to challenge non-firmly adherent THP1. The number of arrested THP1 constantly increased during the accumulation phase and only the one that remained stationary on the endothelial monolayer were manually counted in 5 different FOV along the channel of Ibidi m-Slide VI. FITC channel was used to count fluorescently firmly adhered THP1 cells. Objective 10x, at 40 images per min (1 phase-contrast and 1 FITC), field of view 640 μm × 480 μm, recording time 5.5 min.
10.1186/s13068-016-0531-0 Shear-resistant firm adhesion of JURKAT cells to CONTROL Scrambled-Anti-miR transfected and stimulated with TNFα and IFNγ hCMECD3 cells. JURKAT cells were pulled through the channel over hCMEC/D3 monolayers under low shear (0.5 dyn/cm^2^). After 5 min, flow shear stress was increased (1.5 dyn/cm^2^) to challenge non-firmly adherent JURKAT. The number of arrested JURKAT constantly increased during the accumulation phase and only the one that remained stationary on the endothelial monolayer were manually counted in 5 different FOV along the channel of Ibidi m-Slide VI. FITC channel was used to count fluorescently firmly adhered Jurkat cells. Objective 10x, at 40 images per min (1 phase-contrast and 1 FITC), field of view 640 μm × 480 μm, recording time 5.5 min.
10.1186/s13068-016-0531-0 Shear-resistant firm adhesion of JURKAT cells to CONTROL Scrambled-Anti-miR transfected and stimulated with TNFα and IFNγ hCMECD3 cells. JURKAT cells were pulled through the channel over hCMEC/D3 monolayers under low shear (0.5 dyn/cm^2^). After 5 min, flow shear stress was increased (1.5 dyn/cm^2^) to challenge non-firmly adherent JURKAT. The number of arrested JURKAT constantly increased during the accumulation phase and only the one that remained stationary on the endothelial monolayer were manually counted in 5 different FOV along the channel of Ibidi m-Slide VI. FITC channel was used to count fluorescently firmly adhered Jurkat cells. Objective 10x, at 40 images per min (1 phase-contrast and 1 FITC), field of view 640 μm × 480 μm, recording time 5.5 min.
10.1186/s13068-016-0531-0 Shear-resistant firm adhesion of THP1 cells to CONTROL Scrambled-Anti-miR transfected and stimulated with TNFα and IFNγ hCMECD3 cells. THP1 cells were pulled through the channel over hCMEC/D3 monolayers under low shear (0.5 dyn/cm^2^). After 5 min, flow shear stress was increased (1.5 dyn/cm^2^) to challenge non-firmly adherent THP1. The number of arrested THP1 constantly increased during the accumulation phase and only the one that remained stationary on the endothelial monolayer were manually counted in 5 different FOV along the channel of Ibidi m-Slide VI. FITC channel was used to count fluorescently firmly adhered THP1 cells. Objective 10x, at 40 images per min (1 phase-contrast and 1 FITC), field of view 640 μm × 480 μm, recording time 5.5 min.
10.1186/s13068-016-0531-0 Shear-resistant firm adhesion of THP1 cells to Anti-miR-155 transfected and stimulated with TNFα and IFNγ hCMECD3 cells. THP1 cells were pulled through the channel over hCMEC/D3 monolayers under low shear (0.5 dyn/cm^2^). After 5 min, flow shear stress was increased (1.5 dyn/cm^2^) to challenge non-firmly adherent THP1. The number of arrested THP1 constantly increased during the accumulation phase and only the one that remained stationary on the endothelial monolayer were manually counted in 5 different FOV along the channel of Ibidi m-Slide VI. FITC channel was used to count fluorescently firmly adhered THP1 cells. Objective 10x, at 40 images per min (1 phase-contrast and 1 FITC), field of view 640 μm × 480 μm, recording time 5.5 min.
10.1186/s13068-016-0531-0 Shear-resistant firm adhesion of THP1 cells to Anti-miR-155 transfected and stimulated with TNFα and IFNγ hCMECD3 cells. THP1 cells were pulled through the channel over hCMEC/D3 monolayers under low shear (0.5 dyn/cm^2^). After 5 min, flow shear stress was increased (1.5 dyn/cm^2^) to challenge non-firmly adherent THP1. The number of arrested THP1 constantly increased during the accumulation phase and only the one that remained stationary on the endothelial monolayer were manually counted in 5 different FOV along the channel of Ibidi m-Slide VI. FITC channel was used to count fluorescently firmly adhered THP1 cells. Objective 10x, at 40 images per min (1 phase-contrast and 1 FITC), field of view 640 μm × 480 μm, recording time 5.5 min.

## Abbreviations

BBB: blood–brain barrier
CNS: central nervous system
EAE: experimental autoimmune encephalomyelitis
ICAM1: intercellular adhesion molecule 1
IFNγ: interferon gamma
IKBIP: inhibitor of nuclear factor kappa-B kinase-interacting protein
IKK: IkB kinase
LFA-1: lymphocyte function-associated antigen 1
miRs: microRNAs
MS: multiple sclerosis
NFκB: nuclear factor kappa-light-chain-enhancer of activated B cells
ROCK: rho-associated kinase
VCAM1: vascular cell adhesion molecule 1
VLA-4: very late antigen-4
TNFα: tumor necrosis factor alpha

## References

[1] Nourshargh S, Hordijk PL, Sixt M (2010). Breaching multiple barriers: leukocyte motility through venular walls and the interstitium. Nat Rev Mol Cell Biol.

[2] Ortiz GG, Pacheco-Moises FP, Macias-Islas MA, Flores-Alvarado LJ, Mireles-Ramirez MA, Gonzalez-Renovato ED (2014). Role of the blood–brain barrier in multiple sclerosis. Arch Med Res.

[3] Aird WC (2007). Phenotypic heterogeneity of the endothelium: I. Structure, function, and mechanisms. Circ Res.

[4] Engelhardt B, Ransohoff RM (2012). Capture, crawl, cross: the T cell code to breach the blood–brain barriers. Trends Immunol.

[5] Lucchinetti C, Brück W, Parisi J, Scheithauer B, Rodriguez M, Lassmann H (2000). Heterogeneity of multiple sclerosis lesions: implications for the pathogenesis of demyelination. Annals of Neurology..

[6] Bartel DP (2009). MicroRNAs: target recognition and regulatory functions. Cell.

[7] Thamilarasan M, Koczan D, Hecker M, Paap B, Zettl UK (2012). MicroRNAs in multiple sclerosis and experimental autoimmune encephalomyelitis. Autoimmun Rev.

[8] Ksiazek-Winiarek DJ, Kacperska MJ, Glabinski A (2013). MicroRNAs as novel regulators of neuroinflammation. Mediators Inflamm.

[9] Rom S, Dykstra H, Zuluaga-Ramirez V, Reichenbach NL, Persidsky Y (2015). miR-98 and let-7 g* protect the blood–brain barrier under neuroinflammatory conditions. J Cereb Blood Flow Metab.

[10] Wu D, Cerutti C, Lopez-Ramirez MA, Pryce G, King-Robson J, Simpson JE (2015). Brain endothelial miR-146a negatively modulates T-cell adhesion through repressing multiple targets to inhibit NF-kappaB activation. J Cereb Blood Flow Metab.

[11] Faraoni I, Antonetti FR, Cardone J, Bonmassar E (2009). miR-155 gene: a typical multifunctional microRNA. Biochim Biophys Acta.

[12] Vigorito E, Kohlhaas S, Lu D, Leyland R (2013). miR-155: an ancient regulator of the immune system. Immunol Rev.

[13] Lopez-Ramirez MA, Wu D, Pryce G, Simpson JE, Reijerkerk A, King-Robson J (2014). MicroRNA-155 negatively affects blood–brain barrier function during neuroinflammation. FASEB J..

[14] Weksler BB, Subileau EA, Perriere N, Charneau P, Holloway K, Leveque M, et al. Blood-brain barrier-specific properties of a human adult brain endothelial cell line. FASEB J. 2005:04-3458fje. doi:10.1096/fj.04-3458fje. 10.1096/fj.04-3458fje 16141364

[15] Male DK, Pryce G, Hughes CC (1987). Antigen presentation in brain: MHC induction on brain endothelium and astrocytes compared. Immunology.

[16] Greenwood J, Heasman SJ, Alvarez JI, Prat A, Lyck R, Engelhardt B (2011). Review: leucocyte–endothelial cell crosstalk at the blood–brain barrier: a prerequisite for successful immune cell entry to the brain. Neuropathol Appl Neurobiol.

[17] Steiner O, Coisne C, Cecchelli R, Boscacci R, Deutsch U, Engelhardt B (2010). Differential roles for endothelial ICAM-1, ICAM-2, and VCAM-1 in shear-resistant T cell arrest, polarization, and directed crawling on blood–brain barrier endothelium. J Immunol.

[18] Wu XY, Fan WD, Fang R, Wu GF (2014). Regulation of microRNA-155 in endothelial inflammation by targeting nuclear factor (NF)-kappaB P65. J Cell Biochem.

[19] Selbach M, Schwanhausser B, Thierfelder N, Fang Z, Khanin R, Rajewsky N (2008). Widespread changes in protein synthesis induced by microRNAs. Nature.

[20] Kong W, Yang H, He L, Zhao JJ, Coppola D, Dalton WS (2008). MicroRNA-155 is regulated by the transforming growth factor beta/Smad pathway and contributes to epithelial cell plasticity by targeting RhoA. Mol Cell Biol.

[21] Anwar KN, Fazal F, Malik AB, Rahman A (2004). RhoA/Rho-associated kinase pathway selectively regulates thrombin-induced intercellular adhesion molecule-1 expression in endothelial cells via activation of I kappa B kinase beta and phosphorylation of RelA/p65. J Immunol..

[22] O’Connell RM, Rao DS, Baltimore D (2012). microRNA regulation of inflammatory responses. Annu Rev Immunol.

