# Supplementary material for: MicroRNA-155 contributes to shear-resistant leukocyte adhesion to human brain endothelium in vitro
Source: Fluids Barriers CNS. 2016 May 31;13:8. doi: 10.1186/s12987-016-0032-3 (PMC4888311; doi:10.1186/s12987-016-0032-3)
Supplement: Supplementary file 2 — 10.1186/s13068-016-0531-0 Fig. S1, Table S1, Table S2. [file 12987_2016_32_MOESM2_ESM.docx]

**Supplementary informations**


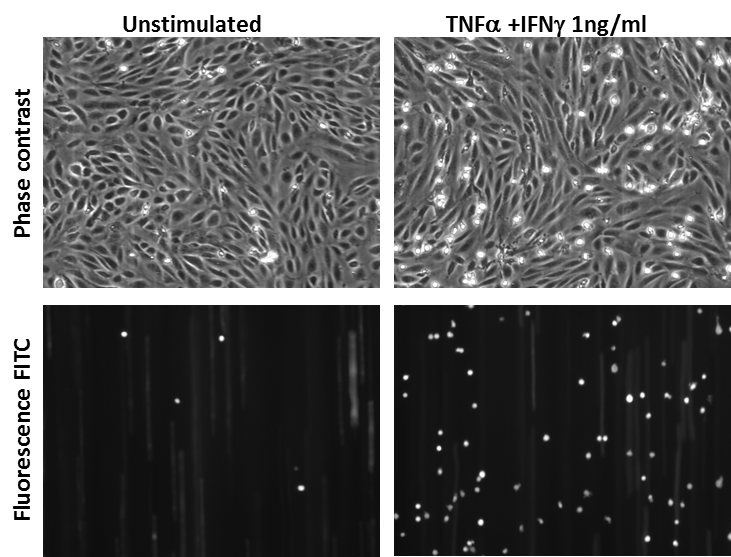


**Fig. S1**: **Representative picture of shear-resistant firmly adhered Jurkat cells to hCMEC/D3 cells. TOP PANELS** Phase contrast pictures of confluent hCMEC/D3 cells stimulated with combination of cytokine (TNFα + IFNγ) at 1 ng/ml for 24 h (RIGHT PANEL) or left unstimulated (LEFT PANEL). **BOTTOM PANELS** Fluorescent pictures (λ_ex_= 495, λ_em_= 521) of firmly adhered Jurkat T cells to hCMEC/D3 cells. Field of view (640 x 480 μm).

| **Parameter** | **Equation** | **Definitions** | **Unit** |
| --- | --- | --- | --- |
| **Shear rate (γ)** | γ = 8v  d | **γ** = Shear rate  **v**= Linear fluid velocity  **d**= Inside diameter of the vessel | [1/s] |
| **Dynamic viscosity (μ)** (mu) | *F* =*  A u*  *y* | The magnitude***F*** of this force is found to be proportional to the speed ***u*** and the area ***A*** of each wall, and inversely proportional to their separation ***y***. | cP(centipoise)  1cP= 1mPa s  =[0.01dyn.s/cm^2^  =  (eta) |
| **Flow rate ()** |  = v⋅  | **V** = Velocity of the blood flowing  **A** = Cross sectional vector of the vessel |  [ml/min] |
| **Shear stress ()** |  =  ⋅ γ | Newtonian fluids flowing upon a planar surface  **** = Shear stress  **γ **= Shear rate  **** = Dynamic viscosity | [dyn/cm^2^  |

**Table S1: Parameters to determine shear stress for vessel blood flow**. Assuming that the vessel is inelastic, cylindrical and straight, and, the blood is a Newtonian fluid and flow is laminar, the Haagen-Poiseuille equation indicates that the shear stress is directly proportional to blood shear rate and inversely proportional to vessel diameter. Shear stress () depends on shear rate (γ) and dynamic viscosity (μ), which are related to the properties of the fluid, and the geometry of the vessel. Blood and water`s dynamic viscosities are 1.2 cP and 1 cP, respectively.

| **Pulled thought Ibidi channel u-slide IV ^0.4^** | **Shear stress** | | **Time (min)** |
| --- | --- | --- | --- |
| **Leukocyte in media** | 0.5 dyn/cm^2^ | 0.28 ml/min | 5 |
| **Media only** | 1.5 dyn/cm^2^ | 0.85 ml/min | 0.5 |

**Table S2: Parameters used for flow based adhesion in Ibidi channel u-slide IV ^0.4.^** Data about the geometry of the chamber can be found at www.ibidi.com.
